# Tranexamic Acid in Dacryocystorhinostomy: A Systematic Review

**DOI:** 10.7759/cureus.104624

**Published:** 2026-03-03

**Authors:** Angeliki Kollatou, Konstantina N Sorkou, Argyrios Tzamalis, Ioannis Tsinopoulos

**Affiliations:** 1 Ocular Surgery, School of Medicine, Aristotle University of Thessaloniki, Thessaloniki, GRC; 2 Department of Ophthalmology, 424 General Military Hospital of Thessaloniki, Thessaloniki, GRC; 3 Second Department of Ophthalmology, Papageorgiou General Hospital/Aristotle University of Thessaloniki, Thessaloniki, GRC

**Keywords:** antifibrinolytics, dacryocystorhinostomy, hemostasis, intraoperative bleeding, nasolacrimal duct obstruction, surgical field clarity, tranexamic acid

## Abstract

Tranexamic acid (TXA) is an antifibrinolytic drug that inhibits the conversion of plasminogen to plasmin, thereby reducing the enzymatic degradation of fibrin and preserving hemostasis across various surgical fields. Dacryocystorhinostomy (DCR) is a frequently performed surgical procedure to manage nasolacrimal duct obstruction (NLDO), in which intraoperative bleeding remains a significant challenge that may impair visualization and prolong operative time. However, the overall impact of TXA in DCR remains uncertain. This systematic review aimed to examine the current research on the role of TXA in DCR, focusing on its impact on intraoperative bleeding, surgical duration, and surgical field clarity. The systematic review was conducted in compliance with PRISMA (Preferred Reporting Items for Systematic reviews and Meta-Analyses) guidelines. PubMed/MEDLINE, Cochrane, and Google Scholar databases were searched up to March 30, 2025, and randomized controlled trials (RCTs) assessing TXA use in DCR were identified. Studies were screened and selected based on predefined eligibility criteria.

Four RCTs involving a total of 286 patients were included. Three studies compared intravenous or topical TXA with normal saline (placebo), and one compared TXA with hydralazine and remifentanil. Two studies, one evaluating intravenous administration and the other topical administration of TXA, demonstrated a statistically significant decrease in both intraoperative hemorrhage and surgical duration. However, one trial reported no significant difference in the assessed outcomes, and the study that compared TXA with alternative agents found greater efficacy with hydralazine and remifentanil. Surgical field clarity and surgeon satisfaction improved with TXA in some cases, but the results were not statistically significant. A low risk of bias was identified in one trial, whereas the remaining studies were judged to raise some concerns, largely attributed to subjective outcome measures.

TXA may be effective in reducing intraoperative hemorrhage and surgical time during DCR, with both topical and intravenous routes demonstrating potential benefits. However, the available evidence remains limited due to small sample sizes, variable outcome measures, and subjective assessments. Further research is required to improve the robustness of the evidence and establish the most effective dosing regimens.

## Introduction and background

Tranexamic acid (TXA) is a synthetic, lysine-derived antifibrinolytic agent first introduced in the 1960s [1]. It acts as a competitive inhibitor of plasminogen activation, thereby preventing its conversion to plasmin and subsequently inhibiting fibrin degradation [2]. TXA reversibly binds to lysine-binding sites on plasminogen, impairing fibrinolysis, promoting clot stability, and is widely recognized as an effective agent for decreasing perioperative bleeding across multiple surgical disciplines [3-5].

Beyond its antifibrinolytic properties, TXA may also exert anti-inflammatory effects by inhibiting plasmin-mediated cytokine release, which has been linked to postoperative erythema, edema, and pain [6,7]. TXA also prevents plasmin-induced platelet activation, thereby maintaining platelet availability for clot formation [2]. Its efficacy in reducing blood loss and transfusion requirements has been extensively reported in trauma, orthopedic, cardiac, plastic, and obstetric surgeries [2,6,8-11]. In ophthalmology, TXA has primarily been used to prevent rebleeding in traumatic hyphema [12], whereas its application in cosmetic surgery has demonstrated benefits in reducing hemorrhage, ecchymosis, and edema [13].

TXA is most commonly administered intravenously, with a half-life of approximately 80 minutes and reaching peak plasma concentrations within one hour [14]. A dose of 10 mg/kg has been shown to achieve effective antifibrinolytic activity, with no additional benefit observed at higher doses [15-17]. Intravenous TXA is routinely used in orthopedic, cardiac, craniofacial, and otolaryngologic procedures at doses ranging from 10 mg/kg to a fixed dose of 1 g [18]. Topical TXA, applied at concentrations ranging from 0.7 to 100 mg/mL, has also demonstrated promising hemostatic efficacy in various surgical settings [17,19,20].

Current evidence does not indicate a significant increase in thrombotic risk with TXA use, although seizures have been reported at high systemic doses [21]. Common adverse effects include orthostatic hypotension and gastrointestinal disturbances, while serious complications are rare, particularly following local administration [21,22]. Nevertheless, TXA is contraindicated in patients with active thromboembolic disease, intravascular coagulation, known hypersensitivity, or subarachnoid hemorrhage because of the risk of cerebral edema and infarction [1,4].

Dacryocystorhinostomy (DCR) is a common oculoplastic procedure performed to bypass nasolacrimal duct obstruction (NLDO) by creating a direct anastomosis between the lacrimal sac and the nasal cavity [23]. NLDO, which has an incidence of 20.24 per 100,000, arises from chronic inflammation, fibrosis, and progressive narrowing of the nasolacrimal duct [24,25]. Clinical manifestations range from partial or complete epiphora to acute dacryocystitis, mucoceles, canalicular obstruction, and dacryolith formation [25]. Secondary acquired NLDO and persistent congenital obstruction also represent established indications for DCR [26,27].

The main surgical approaches include external (EX-), endoscopic (EN-), and transcanalicular (TC-) DCR [24].EX-DCR has traditionally been considered the gold standard, with reported success rates ranging from 85 to 99% [28,29]. However, it is associated with potential complications, including hemorrhage, infection, scarring, and disruption of medial canthal anatomy [30]. In recent years, EN-DCR has been increasingly utilized as an alternative, with a growing body of literature demonstrating comparable outcomes to EX-DCR and highlighting its advantages, including the avoidance of external scars and the simultaneous management of intranasal pathology [31]. EN-DCR has gained popularity over the past two decades and is now widely performed in many centers as part of routine practice [32]. TC-DCR also offers advantages such as reduced operative time and minimal tissue disruption [33].

Despite technical advances, intraoperative hemorrhage remains a major challenge, as it can obscure the limited surgical field, prolong operative time, and compromise surgical outcomes [34-36]. Even moderate bleeding in DCR may significantly impair visualization within the confined operative space, potentially affecting osteotomy precision and long-term patency [34-36]. Postoperative complications include bleeding, edema, granulation tissue formation, canalicular injury, and intranasal synechiae [24]. Bleeding during DCR most commonly arises from the richly vascularized nasal mucosa and the lacrimal sac fossa, where even limited hemorrhage may rapidly obscure the narrow operative field [24]. Although multiple strategies are employed to minimize bleeding, including vasoconstrictors, intranasal packing, and meticulous surgical technique, unexpected hemorrhage may still occur, highlighting the need for adjunctive hemostatic approaches [34,37].

Recent studies have investigated the role of TXA in reducing intraoperative bleeding and perioperative complications in oculoplastic procedures, including DCR. However, the overall impact of TXA in DCR remains uncertain, as available randomized trials are small and report heterogeneous and sometimes conflicting findings across different administration routes and study designs. While quantitative synthesis exists in the literature, important questions remain regarding the procedure-specific efficacy of TXA in DCR. A focused qualitative evaluation of study design, heterogeneity, and clinical relevance is therefore important. Given the procedure-specific technical demands of DCR, a focused appraisal of the available randomized evidence may provide clinically useful clarification. This systematic review aims to critically assess the available evidence on TXA use in DCR, specifically evaluating its impact on intraoperative bleeding, surgical field quality, operative duration, and perioperative complications.

## Review

Materials and methods

A systematic review was conducted in accordance with the Preferred Reporting Items for Systematic Reviews and Meta-Analyses (PRISMA) guidelines [38]. No review protocol was prospectively registered.

Eligibility Criteria

Randomized controlled trials (RCTs) published in English were eligible for inclusion, with no restrictions on publication year or geographic location. Systematic reviews and case reports were excluded. Eligible studies included patients with nasolacrimal duct obstruction undergoing external or internal DCR. The intervention of interest was the administration of TXA, either intravenously or topically. Studies were required to report data on at least one predefined outcome, including intraoperative bleeding or operative duration. No limitations were placed on follow-up duration. Both adult and pediatric populations were considered eligible if reported, while quasi-randomized studies were excluded.

Data Sources

PubMed/MEDLINE, the Cochrane Database of Systematic Reviews, and Google Scholar were searched to identify eligible studies. Reference lists of included articles were also manually screened for additional relevant publications.

​*Search Strategy*

A comprehensive and structured search strategy was developed using Medical Subject Headings (MeSH) and free-text terms to identify studies evaluating TXA in patients undergoing DCR and its effectiveness in reducing intraoperative bleeding. The search included the terms “tranexamic acid,” “TXA,” “dacryocystorhinostomy,” “DCR,” “external dacryocystorhinostomy,” and “internal dacryocystorhinostomy.” Synonyms and alternative spellings of these terms were also considered. An example of the PubMed search syntax was: (“tranexamic acid” OR TXA) AND (“dacryocystorhinostomy” OR DCR OR “external dacryocystorhinostomy” OR “internal dacryocystorhinostomy”). The date of the final search was March 30, 2025. The search was limited to randomized controlled trials involving human subjects. For Google Scholar, results were screened in order of relevance until no additional eligible studies were identified. The core PubMed search syntax is provided above to allow reproducibility.

Study Selection and Data Collection

Study selection was performed by a single reviewer using predefined eligibility criteria and a standardized screening process. Duplicate records were removed, followed by screening of titles and abstracts, and then full-text review of potentially eligible studies. Duplicates were identified and removed through manual verification. The reviewer was not blinded to journal titles or author information. A predefined screening framework was applied consistently across all records to reduce the risk of selection bias.

Data Extraction

Data were extracted by a single reviewer using a standardized and predefined approach. A structured data extraction framework was applied to maintain consistency across studies. The predefined data items included study characteristics (authors, year, study design, sample size, and country of origin), participant demographics and eligibility criteria, details of the intervention (TXA route, dosage, timing, type of DCR, anesthesia, and follow-up duration), and reported outcomes. Predefined outcomes of interest included intraoperative hemorrhage, operative time, and surgical field visualization. When available, reported p-values were reviewed to determine statistical significance.

Risk-of-Bias Assessment

The methodological quality of included studies was assessed using the Cochrane Risk of Bias tool for randomized trials (RoB 2) [39]. Each study was evaluated across five domains: the randomization process, deviations from intended interventions, missing outcome data, outcome measurement, and selective reporting. Each domain was rated as low risk, some concerns, or high risk of bias, resulting in an overall risk-of-bias assessment for each study. The RoB 2 signaling questions were applied according to Cochrane guidance to inform domain-level judgments.

Synthesis Methods

Given the limited number of included studies and substantial clinical and methodological heterogeneity in study design, TXA administration protocols, outcome definitions, and comparator interventions, a meta-analysis was considered inappropriate, and a qualitative synthesis was conducted instead. Prespecified summary effect measures were not calculated because quantitative pooling was not undertaken. Continuous outcomes were interpreted descriptively based on reported group means and the observed direction of effect. Due to the small number of included studies, formal assessment of reporting bias and certainty of evidence (e.g., GRADE) was not performed.

Results

Study Selection

**Figure 1:**
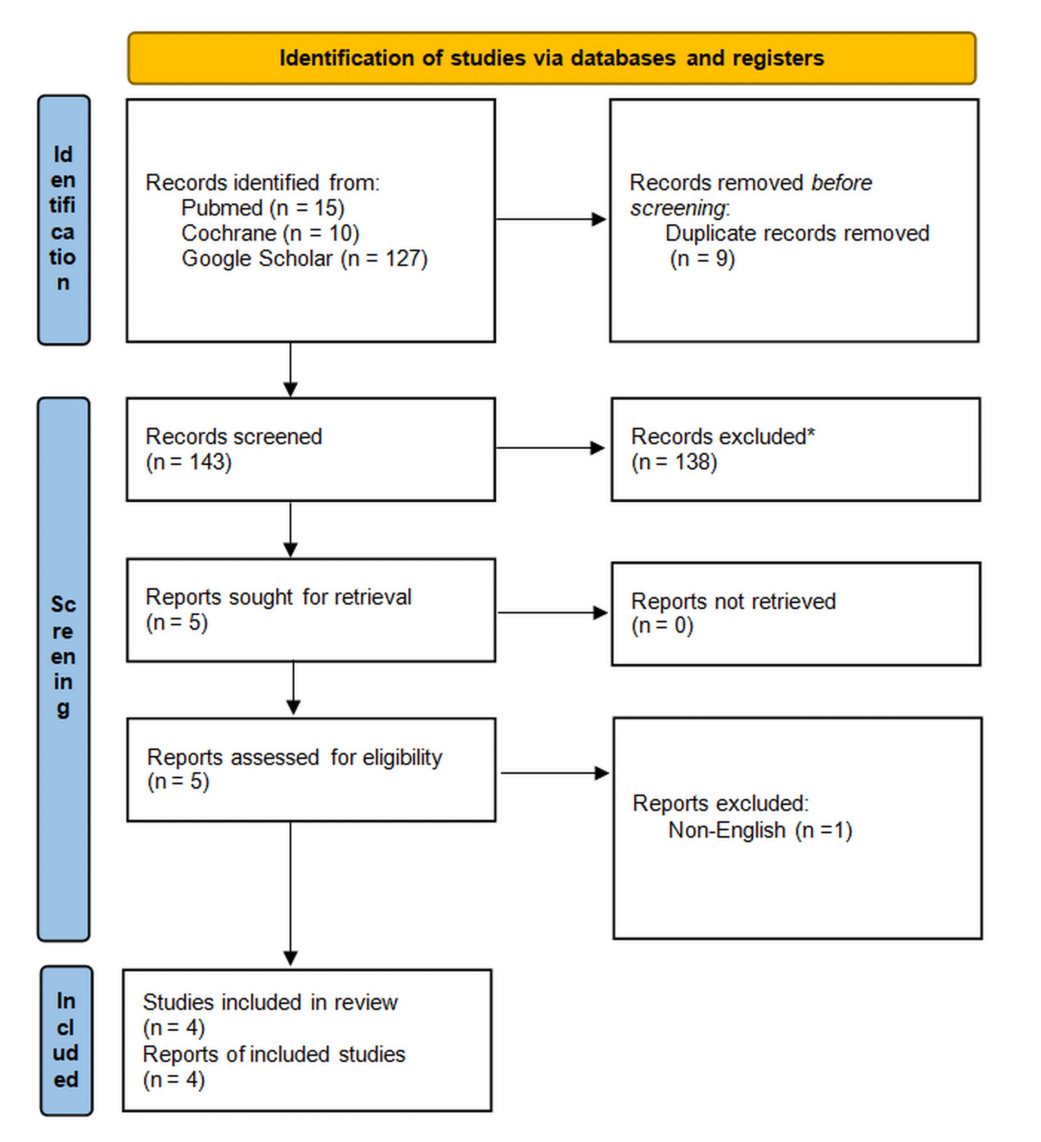
PRISMA 2020 flow diagram depicting the study selection process ^*^Records excluded during the screening phase based on titles and abstracts that did not meet the inclusion criteria PRISMA Preferred Reporting Items for Systematic Reviews and Meta-Analysis

The literature search conducted in PubMed/MEDLINE, the Cochrane Database, and Google Scholar yielded a total of 152 records. No automation tools were used. After removing nine duplicate records, 138 studies were excluded during title and abstract screening due to irrelevance. Five full-text articles were reviewed for eligibility. One full-text article was excluded due to being published in a language other than English. Ultimately, four randomized controlled trials met the inclusion criteria and were included in this systematic review [40-43]. The PRISMA flow diagram illustrating the study selection process is presented in Figure 1 [38]. 

Study Characteristics

A total of four randomized controlled trials were included. The main characteristics of the selected studies are summarized in Table 1. In most trials [40-42], participants were randomized to receive TXA or placebo, whereas one study [43] compared TXA with alternative hypotensive agents. TXA was administered intravenously in three studies [40,42,43], while one study employed topical administration using gauze soaked with TXA and adrenaline [41]. Additional details regarding patient demographics, anesthetic protocols, TXA dosage, and reported outcomes are presented in Table 1.

**Table 1:** Characteristics of the studies included in the systematic review

Study		Procedure		Anesthesia technique	Intervention		Control group/comparators		Total patients	Number of patients in inter/control			Age (years), inter/control		Female %, inter/control	Outcomes available		Key findings	
Alam et al. (2022) [40]		External dacryocystorhinostomy		No general anesthesia	TXA IV 1g (30 minutes before surgery)		Normal saline (placebo)		96	51/45			49.63/49.58		47.1/60	Intraoperative bleeding volume, surgical time, field visibility, and need for nasal packing		No statistically significant differences in the amount of blood loss, surgical time, surgical field, and need for nasal packing between the two groups	
Salamah et al. (2023) [41]		External dacryocystorhinostomy		General anesthesia	Gauze soaked with combined TXA (100 mg/ml) and epinephrine 1:200,000 (for 2 minutes)		Gauze soaked only with epinephrine 1: 200,000		30	15/15			39.9/42		53.3/66.7	Intraoperative bleeding volume, number of used gauzes, and the surgeon’s assessment of satisfaction, surgery time, and field visibility		Significant difference in blood loss, number of used gauzes, and surgical time. No significant difference in field clarity and surgeon satisfaction	
Sharifi et al. (2024) [42]		External dacryocystorhinostomy		General anesthesia	TXA IV 10 mg/kg with a maximum dose of 1g (30 minutes before surgery)		Normal saline (placebo)		70	35/35			55.46/58.06		40/45.7	Intraoperative bleeding volume and surgical time		Significant difference between the two groups in both surgical time and bleeding volume	
Moradi Farsani et al. (2022) [43]		External dacryocystorhinostomy		General anesthesia	TXA IV 10 mg/kg with a maximum dose of 1 g, prescribed in 100 cc dextrose water 5% serum (15 minutes before surgery)		Hydralazine, remifentanil		90	30/30/30			51.3/53.9/46.2		50/50/50	Blood loss and surgeon’s satisfaction, surgery time, and hemodynamic variables		Blood loss significantly lower in the remifentanil and hydralazine groups compared with the TXA group. No significant difference in all the other parameters in all three groups	

Risk of Bias in Studies

Evaluation of bias was performed in accordance with the Cochrane RoB 2 [39] and is presented in Table 2. Sharifi et al.'s study [42] was judged to be at low risk across all domains due to clear allocation concealment, double-blinding, and objective outcome measurement. The remaining studies [40,41,43] were rated as having "some concerns" due to subjective outcome components such as surgical field grading and surgeon satisfaction. No studies had missing data or evidence of selective reporting of results. 

**Table 2:** Risk-of-bias assessment of the included studies

Study	Randomization process	Deviations from intended interventions	Missing outcome data	Measurement of the reported result	Selection of the reported result	Overall
Alam et al. (2022) [40]	Low risk	Low risk	Low risk	Some concerns	Low risk	Some concerns
Salamah et al. (2023) [41]	Low risk	Low risk	Low risk	Some concerns	Low risk	Some concerns
Sharifi et al. (2024) [42]	Low risk	Low risk	Low risk	Low risk	Low risk	Low risk
Moradi Farsani et al. (2022) [43]	Low risk	Low risk	Low risk	Some concerns	Low risk	Some concerns

Intraoperative Bleeding

The primary endpoint evaluated in this review was intraoperative blood loss. All studies reported bleeding as mean ± standard deviation (SD). In two studies, Salamah et al. [41] and Sharifi et al. [42], there was a significant reduction in blood loss as the amount of bleeding was 29.4 ± 17.1 ml in the TXA group vs. 49.1 ± 18.1 ml in the control group, with a p-value of 0.005 in the first one (topical TXA), and 47.74 ± 60 ml in the intervention group vs. 70.66 ± 48.19 ml in the control group, with a p-value of <0.001 in the second (intravenous administration of TXA). However, Alam et al. [40] reported no statistically significant difference, as the average volume of blood lost in the TXA group was 88.63 ± 69.34 ml, compared to 88.89 ± 51.93 ml in the placebo group (p=0.984). Moreover, postoperative nasal packing was administered to 28 patients in both groups (54.9% vs. 62.2%) to control bleeding after surgery, with no significant difference (p=0.471). Moradi Farsani et al. [43] described a significantly lower average hemorrhage volume that was observed in the remifentanil and hydralazine groups relative to the TXA group (TXA group: 146.83 ± 91 ml, remifentanil group: 77.6 ± 52.1 ml, hydralazine group: 80.0 ± 48.7 ml).

Surgical Time

Both Salamah et al. [41] and Sharifi et al. [42] documented a significant reduction in the duration of surgery. Salamah et al. [41] reported that the operation time in the TXA group was 36 ± 8.7 minutes vs. 46.1 ± 11.7 minutes in the control group (p=0.01). Sharifi et al. [42] supported these outcomes, reporting a p-value <0.001, as the length of the surgical procedure was 26.03 ± 10.5 minutes in the intervention group and 37.74 ± 9.52 minutes in the control group. Conversely, Alam et al. [40] presented no statistically significant difference in surgery time (48.43 ± 20.01 min vs. 53.38 ± 19.8 min, p=0.228). Moradi Farsani et al. [43] reported higher operation time in the TXA group, in comparison to the other three studies (72.4±15.7 minutes). No notable statistical differences were observed among the three groups (64.1 ± 18.7 minutes in the hydralazine group, 70.5 ± 15.1 minutes in the remifentanil group).

Surgical Field Quality and Surgeon Satisfaction

Surgical field clarity was assessed in two studies [40,41], which reported this parameter as part of their outcome evaluation. Intravenous administration in the trial presented by Alam et al. [40] indicated that enhanced visibility of the operative field with limited hemorrhage was observed in 25 patients (49%) in the study group and 16 patients (35.6%) in the control group. In contrast, the surgical field was obstructed in 17 patients (33.3%) in the study group and 12 patients (26.7%) in the control group. However, there was no statistical significance (p=0.084). Correspondingly, the topical route (gauze saturated with adrenaline and TXA) in the trial by Salamah et al. [41] reported that, despite an improvement in surgical field visibility, the finding lacked statistical relevance (p=0.081).

Furthermore, two studies [41,43] evaluated the surgeon’s satisfaction concerning the visualization of the operative area and procedural simplicity. Although surgeon satisfaction was high in the topical TXA group as reported by Salamah et al. [41], the observed difference did not reach statistical significance (p=0.087). The trial presented by Moradi Farsani et al. [43] investigated the degree of surgeons’ satisfaction between the three groups (TXA, hydralazine, and remifentanil), without finding any significant difference.

Discussion

Principal Findings

DCR is a frequently performed oculoplastic procedure in which intraoperative hemorrhage is the main surgical concern, as it can significantly impair the surgeon’s visual field during surgery [36]. The extent of bleeding not only affects the technical ease of the procedure but can also substantially influence surgical outcomes, particularly in cases of excessive hemorrhage [36]. This systematic review included four RCTs that evaluated the impact of TXA, primarily focusing on reducing bleeding and shortening operative time during EX-DCR. The studies differed in the route of TXA administration, type of anesthesia, and comparator interventions. The results suggest that TXA may be an effective agent in DCR surgery, particularly when administered appropriately and in specific clinical settings. However, the overall strength of evidence remains limited due to the small number of randomized trials, small sample sizes, and methodological heterogeneity.

Among the included studies, Sharifi et al. [42] provided the strongest evidence supporting TXA use. When administered intravenously during general anesthesia, TXA significantly reduced intraoperative bleeding and shortened surgical time. This study was also evaluated as having a low risk of bias across all domains, thereby strengthening the reliability of its findings. The use of objective outcome measures and a robust randomization process further supports the conclusion that systemic TXA under general anesthesia may be beneficial in DCR.

Conversely, Alam et al. [40] reported no significant decrease in bleeding after TXA was administered intravenously under local anesthesia. They observed a higher volume of intraoperative bleeding in both study groups compared with that reported in the trials conducted by Sharifi et al. [42] and Salamah et al. [41], which may be attributed to a prolonged surgical duration. Similarly, Moradi Farsani et al. [43] documented a substantially greater bleeding volume (146.83 ± 91 ml), potentially due to the extended duration of the DCR and the specific drug administration protocol. In their study, TXA was delivered intravenously over 15 minutes before surgery, which may not have allowed adequate time for the drug to achieve peak serum concentration at the onset of the procedure.

Sharifi et al. [42] further proposed that the differences observed between their results and those reported by Alam et al. [40] could be attributed to variations in anesthesia techniques and the surgeons’ levels of experience, both of which are known to influence the degree of bleeding in DCR surgeries. Salamah et al. [41] evaluated topical TXA combined with epinephrine compared with epinephrine alone. The combination resulted in significantly reduced blood loss and surgical time, suggesting an effective and potentially safer alternative to systemic administration. These findings indicate that topical TXA, particularly in combination with vasoconstrictors, may have a unique role in DCR procedures and highlight the need for further investigation into this route.

Comparison With Existing Literature

These findings partially correspond with those reported in a recent systematic review and meta-analysis of nine RCTs, including 827 patients undergoing oculoplastic surgeries [44]. That review concluded that TXA, whether administered intravenously or subcutaneously, did not demonstrate a statistically meaningful decline in intraoperative blood loss overall. However, a subgroup analysis suggested a decrease in bleeding in DCR surgeries when TXA was administered intravenously, although this trend was not statistically significant. This is consistent with the variability observed in this review, where Sharifi et al. [42] reported a significant reduction in bleeding, while Alam et al. [40] found no such benefit.

Moreover, the meta-analysis found no clear improvement in surgical duration with TXA use, whereas in the present review, only two studies, Sharifi et al. [42] and Salamah et al. [41], observed a notable decline in operative time. Regarding surgeon satisfaction and field clarity, both the current review and the meta-analysis identified a general improvement with TXA use, but without statistically significant differences. This is likely due to the subjective nature of the assessments and the variability in scales used across studies.

Clinical Implications

Although direct evidence regarding TXA use in DCR remains relatively limited, findings from related oculoplastic procedures provide useful contextual support. A split-face study evaluating subcutaneous TXA in eyelid surgery demonstrated significantly reduced postoperative ecchymosis and edema at early follow-up points, suggesting a beneficial local effect on periocular tissues [37]. Similarly, a randomized controlled trial comparing intravenous TXA, subcutaneous TXA, and no TXA in upper eyelid blepharoplasty reported improved control of intraoperative bleeding and reduced postoperative bruising and swelling in both TXA groups, with intravenous administration showing superior hemostatic efficacy [18].

More recently, a systematic review and meta-analysis of randomized trials focusing on external DCR reported a significant reduction in intraoperative bleeding and improved surgeon satisfaction with TXA use, while effects on operative time were less consistent [45]. These findings are consistent with the broader oculoplastic literature and support a potential role for TXA in optimizing the surgical field during DCR. Nevertheless, heterogeneity in administration routes and outcome reporting highlights the need for further well-designed trials directly comparing topical and systemic TXA in DCR to better define optimal protocols and safety profiles. Therefore, the clinical significance of the observed effect sizes must be interpreted cautiously in routine practice.

Strengths and Limitations

This review also has several strengths, including a focused evaluation of randomized evidence specifically in DCR and a structured risk of bias assessment. Several limitations should be considered when interpreting the findings of this systematic review. All studies were assessed by a single reviewer and included relatively small sample sizes, which may limit statistical power and generalizability. Heterogeneity in TXA dosing protocols and outcome measures, along with reliance on subjective assessments such as surgical field visibility and surgeon satisfaction, further complicates comparisons and may introduce bias. In addition, Embase and clinical trial registries were not searched due to institutional access limitations, which may have resulted in the omission of potentially eligible studies. Furthermore, the restriction to English language publications may have introduced language bias, although only one potentially relevant non-English study was identified during screening. Additionally, long-term follow-up on postoperative bleeding or adverse events was lacking.

Future Directions

Future research should prioritize conducting larger, multicenter randomized trials with standardized TXA protocols, direct comparisons of topical and systemic administration, objective quantification of intraoperative bleeding, validated subjective assessment tools, and comprehensive evaluation of cost-effectiveness and safety outcomes to generate more reliable guidance for clinical practice.

## Conclusions

The findings of this systematic review suggest that TXA may reduce intraoperative blood loss and operative time in DCR, with a possible improvement in surgical field quality. However, the current evidence base is limited by the small number of randomized trials, small sample sizes, and methodological heterogeneity. At present, TXA appears promising in DCR. However, the available evidence remains limited and insufficient to support its universal routine use. Further well-designed, adequately powered randomized trials with standardized TXA protocols and objective outcome measures are required to better define its role in routine clinical practice.
